# Protective Effects of Thymoquinone against Methotrexate-Induced Germ Cell Apoptosis in Male Mice

**DOI:** 10.22074/ijfs.2015.4614

**Published:** 2015-12-23

**Authors:** Fatemeh Sheikhbahaei, Mozafar Khazaei, Arezou Rabzia, Kamran Mansouri, Ali Ghanbari

**Affiliations:** 1 Fertility and Infertility Research Center, Kermanshah University of Medical Sciences, Kermanshah, Iran; 2 Medical Biology Research Center, Kermanshah University of Medical Sciences, Kermanshah, Iran

**Keywords:** Apoptosis, Methotrexate, Thymoquinone, Mice, Germ Cells

## Abstract

**Background:**

Toxic effects of anti-cancer and other drugs on the normal tissues could be reduced by the herbal plants and their fractions. This study investigated the protective effect of thymoquinone (TQ) as a fraction of Nigella sativa on methotrexate (MTX)- induced germ cell apoptosis in male mice.

**Materials and Methods:**

In this experimental study, thirty male Balb/c mice were divided randomly into 5 groups (n=6). A single dose of MTX (20 mg/kg) and different concentrations of TQ were administrated for 4 consecutive days. Terminal deoxynucleotidyl transferase dUTP nick end labeling (TUNEL) assay was performed on paraffin embedded tissue sections to analysis the occurrence of apoptosis in the testis. Reverse transcription polymerase chain reaction (RT-PCR) of apoptosis-related genes was performed with RNA extracted from testes of the mice. Statistical analysis was done using one-way ANOVA.

**Results:**

In the MTX group, there was a significant increase in morphologic sign of germ cell degeneration of tubules (48 ± 0.6%), apoptotic index (AI; 2.3 ± 0.6%), as well as mRNA expression of *p53* (P=0.008), *caspase 8* (P=0.002), *caspase 3* (P=0.005), *caspase 9* (P=0.000), bax (P=0.004) and the ratio of *bax/bcl-2* (P=0.000), whereas there was an decrease in the expression of bcl-2 (P=0.003), as compared to control group. In MTX+TQ groups, the data showed that different concentrations of TQ could improve the harmful effects caused by the MTX. The best protective effects were achieved in MTX+TQ (10 mg/kg).

**Conclusion:**

TQ protects testicular germ cell against MTX-induced apoptosis by affecting related genes regulation.

## Introduction

The use of methotrexate (MTX) drug , a widely used folate antagonist, has been limited based on the occurrence of short-and long-term toxicity (1,3). The genotoxic effects of MTX have been also shown in both somatic and germ cells (4). Only a single exposure to high dose of MTX induces germ cell toxicity in mice that can be transmitted to the next generation, explaining the hazardous administration of this drug (5). 

Nigella sativa (black seed) plant has been investigated for its antioxidant, anti-inflammatory and anticancer activities in both *in vitro* and *in vivo* models since 1960s (6). Toxicological studies have shown that thymoquinone (TQ) as the main active component of N. sativa might have a protective effect against hepatotoxicity and nephrotoxicity induced by either chemicals or diseases (7,8). In addition, this quinone compound was found to exhibit anticancer activity through the modulation of multiple molecular targets, including *p53, PTEN, STAT3*, and *PPAR*, activation of *caspases* and generation of reactive oxygen species (ROS) (6). 

Gkce et al. (9) suggested that TQ may decrease the destructive effects of MTX on testicular tissue. Further, Badary et al. (10) have shown that TQ has strong antioxidant activities through scavenging ability of different free radicals in an *in vitro* model. 

Although germ cell toxicity of MTX and protective effects of TQ against hazardous agents have been shown previously, the involvement of apoptosis and its related genes in this issue have not been demonstrated. In this regards, this study was conducted to evaluate the protective effect of TQ against MTXinduced germ cell toxicity of mice testis. The occurrence of apoptosis in seminiferous tubules was shown using terminal deoxynucleotidyl transferase dUTP nick end labeling (TUNEL) assay, and the related genes were characterized by reverse transcription polymerase chain reaction (RT-PCR). 

## Materials and Methods

### Experimental design

In this experimental study, thirty male Balb/c mice aged 10 weeks (30 ± 2 g) were obtained from a closed bred colony at Kermanshah University of Medical Sciences, Kermanshah, Iran. The animals received care as recommended by the Ethics Research Committee of the Kermanshah University of Medical Sciences (EC/KNRC/90-4) in accordance with the internationally accepted principles for laboratory animal use and care, as found in the European Community guidelines (EEC Directive of 1986; 86/609/EEC) or US guidelines (NIH publication #85-23, revised in 1985). The mice were maintained on a regular diet and water at a 12:12 hour light/dark cycle at 23˚C ± 2˚C. Experiment was started after one week adaptation. 

The animals were divided randomly into following 5 groups (n=6): i. Control group receiving dimethyl sulfoxide (DMSO, 1:1000) in normal saline, ii. Experimental group (E1) receiving only an intraperitoneal single dose injection of MTX (20 mg/kg; Sigma Al- drich, USA), iii. Experimental groups (E2-E4) receiving an intraperitoneal injection of MTX (20 mg/kg) plus TQ (Sigma Aldrich, USA) in different concentrations of 2 mg/kg (E2), 10 mg/kg (E3), and 20 mg/kg (E4) for 4 consecutive days (8). On the day five, the mice were sacrificed by cervical dislocation. 

### Terminal deoxynucleotidyl transferase dUTP nick end labeling assay

Apoptosis was assessed by TUNEL assay using In Situ Cell Death Detection Kit (Roche Diagnostics Deutschland GmbH, Germany). After deparfination with xylene, 5 µm sections prepared by rotary microtome. Then, the sections were rehydrated through a series of ethanol solutions and washed in deionized water. Nuclei in the tissue sections were stripped from protein by incubating with 50 µl of proteinase K (10 mg/ml) for 20 minutes at room temperature. 

After washing twice with sterile phosphate-buffered saline (PBS) for 10 minutes, the slides were incubated with TUNEL reaction mixture in a humidified chamber at 37˚C for 60 minutes, followed by rinsing three times with PBS for 10 minutes. The sections were counterstained with propidium iodide (PI) solution diluted to 1 µg/ml in PBS (15 minutes), and then washed in deionized water for 5 minutes. Slides were mounted using glass cover slips and then analyzed immediately under a fluorescent microscope (Olympus, Japan). Apoptotic index (AI) was calculated by dividing the number of TUNEL positive cells to total number of the cells in randomly selected fields, and the result was multiplied by 100 (11). 

### Reverse transcription–polymerase chain reaction analysis

RNA was extracted from testes tissues using the RNAeasy Plus Mini Kit (Qiagen, Germany), including a gDNA Eliminator column to avoid DNase digestion and a RNeasy Mini Spin columns to purify RNA samples. Total RNA (≤1 µg) was reverse transcribed using a poly T tail primer included in the One Step RT-PCR Kit (Qiagen, Germany). cDNA was amplified according to the manufacturer’s instructions. Primer pairs, amplicon sizes, and annealing times are shown in table 1. Cycle conditions were as follows: denaturation at 95˚C for 15 minutes that was followed by 30 cycles at 94˚C for 60 seconds, annealing at 58˚C to 60˚C for 60 seconds, and elongation at 72˚C for 60 seconds, with a final cycle at 72˚C for 10 minutes. Experiments were performed in triplicate to ensure reproducibility. 

Products were electrophoresed on a 1.5% agarose gel. Gels were stained with ethidium bromide (10 µg/ ml) and photographed on an ultraviolet (UV) transilluminator (UVIdoc, Uvitec, UK). Gel images were analyzed using the UV image (UVI) band map program (Uvitec, UK). Primers characteristics are listed in table 1. 

RT–PCR values were presented as a ratio of the specified gene signal divided by the glyceraldehyde- 3-phosphate dehydrogenase (gapdh) signal. RT-PCR was performed as three individual replicates (12). 

### Statistical analysis

All data were analyzed by one-way ANOVA followed by Tukey’s test using SPSS (SPSS Inc., USA) software. Results are expressed as the mean ± SEM, and P<0.05 was considered significant. 

### Results

#### Terminal deoxynucleotidyl transferase dUTP nick end labeling assay

**Fig.1:**
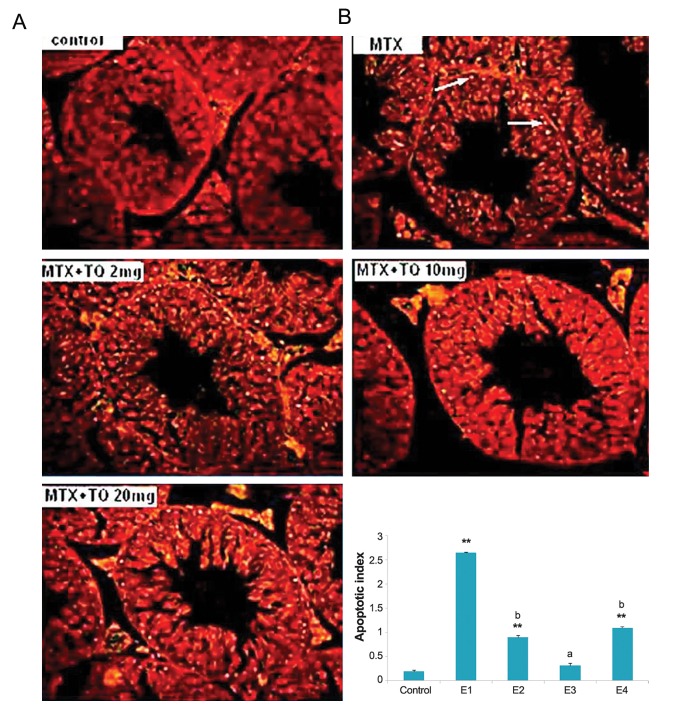
The apoptosis inducing effect of methotrexate (MTX) (20 mg/kg) and different doses of thymoquinone (TQ) on testis of mice. A. Images by a fluorescent microscopy indicating terminal deoxynucleotidyl transferase dUTP nick end labeling (TUNEL) staining of mice testicular sections that are counterstained with propidium iodide (PI). Apoptotic cells show bright fluorescence nuclei indicated by arrowheads (magnifications: ×160) and B. Percent of TUNEL positive cells (AI). The mice were grouped as Control, MTX (E1), MTX+TQ 2 mg/kg (E2), MTX+TQ 10 mg/kg (E3), MTX+TQ 20 mg/kg (E4). **; P<0.001 compare to control group, a; P <0.01 and b; P<0.05 compare to MTX group.

TUNEL assay was performed in the testis to ascertain the mode of cell death by MTX and TQ. In this regards, cross sections of seminiferous tubules were stained with TUNEL dye and analyzed. The AI was quantitatively higher in the MTX-treated group (E1, 2.3 ± 0.4) than the control group (0.2 ± 0.6, P=0.000). Treatment of TQ markedly reduced the reactivity and the number of apoptotic cells (Fig .1A,B). These data indicated that inhibition of germ cell apoptosis is the another action of TQ in basal portion of seminiferous tubules. 

### The expression of markers

**Fig.2:**
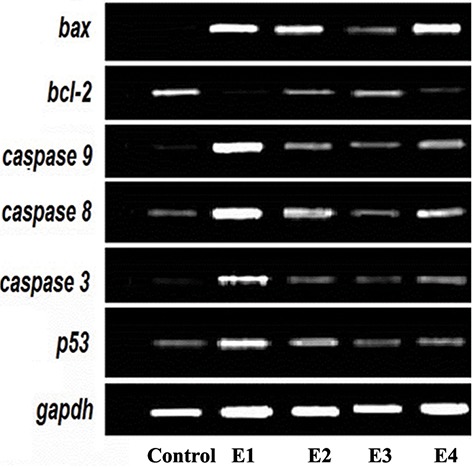
Temporal expression pattern of genes in testis of mice treated with MTX plus different concentrations of TQ using by reverse transcription polymerase chain reaction (RT-PCR) in 4 experimental groups (E1-E4). MTX; Methotrexate and TQ; Thymoquinone.

**Fig.3:**
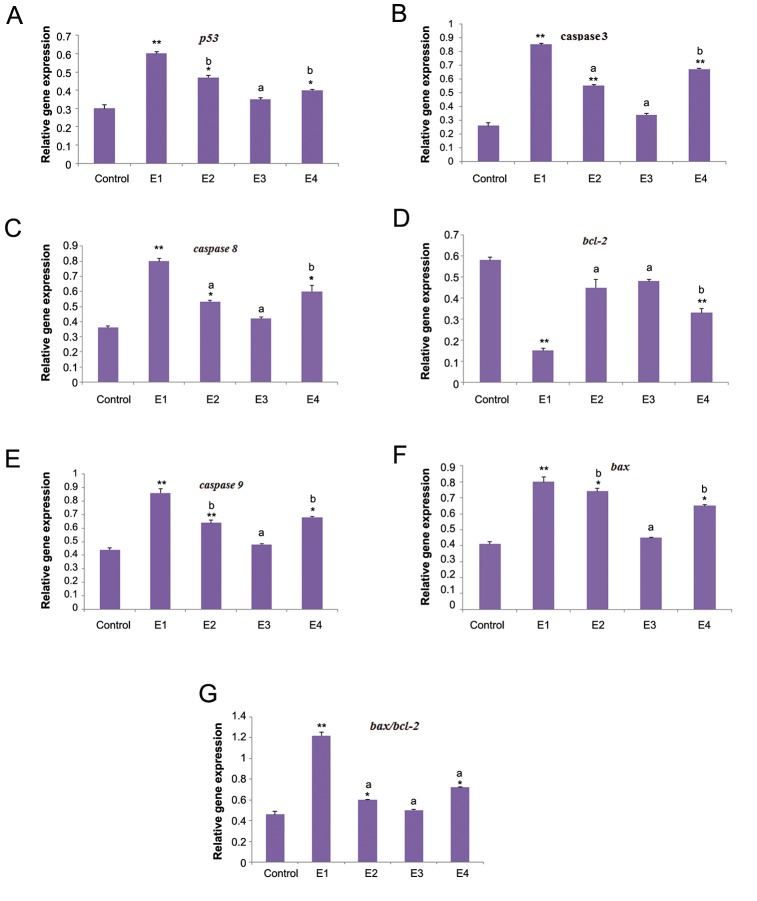
Expression of related genes of apoptosis in testis of mice treated with MTX plus different concentrations of TQ using RT-PCR in 4 experimental groups (E1-E4). The differences between groups are presented by ANOVA. All the values are expressed as mean ± SEM. MTX; Methotrexate, TQ; Thymoquinone, RT-PCR; Reverse transcription polymerase chain reaction, **; P<0.001, *; P<0.01 compare to control group, a; P <0.01 and b; P<0.05 compare to MTX group.

The expression levels of markers and profiles of the relative expression levels are shown in figures 2 and 3. In the E2 group, there was a significant decrease in expression of *bcl2* (P=0.003), whereas there was a significant increase in the expression levels of other markers, including *p53* (P=0.008), *caspase 3* (P=0.005), *caspase 8* (P=0.002), *caspase 9* (P=0.000), and bax (P=0.004). The ratio of *bax/bcl2* also increased in E1 group (P=0.000). The expression levels of these markers in MTX+TQ groups (E2-E4) were between the related values of E1 and control groups. 

Furthermore, the expression level of *bcl2* significantly decreased in E2 (P=0.002) and E3 groups (P=0.005). 

**Table 1:** Primers used for RT-PCR applied for apoptosis-related gene expression in testis of mice


Gene	Primer sequence	Annealing temperature	Size(bp)

*gapdh*	F:ACCTCAACTACATGGTCTAC	58	801
	R:TTGTCATTGAGAGCAATGCC		
*p53*	F:CATCATCACGCTGGAAGACTC	58	378
	R:TCAGCTCTCGGAACATCTC		
*bax*	F:GCTGATGGCAACTTCAACTG	56	280
	R:GATCAGCTCGGGCACTTTAG		
*bcl-2*	F:AGCGTCAACAGGGAGATGTC	58	389
	R:TTCCACAAAGGCATCCCAGC		
*caspase 3*	F:TGTCATCTCGCTCTGGTACG	58	419
	R:CCCTTTCTGCCTGTCTTCTG		
*caspase 8*	F:ACAATGCCCAGATTTCTCCCTAC	59	437
	R:CAGACAGTATCCCCGAGGTTTG		
*caspase 9*	F:CATCCTTGTGTCCTACTCCACC	59	214
	R:CAGCTTTTTCCGGAGGAAGT		


## Discussion

In the present study, the percentage of apoptotic cells was found to be increased after MTX treatment. It has been further reported that MTX induced apoptosis in the hepatocytes of rats, which can be attributed to the nucleotide pool imbalance or the repression of the cJun N-terminal kinase (JNK) activity and upregulation of *p53* and *p21* (13). Consistent with these results, our study showed that administration of MTX induced testicular damage characterized by seminiferous tubule degeneration and apoptosis of germ cells via both *p53* and *Bax/Bcl-2* pathways. 

The release of cytochrome c from mitochondria has been indicated to be a critical step in the activation of the caspase protease cascade. Caspases trigger a cascade of proteolytic cleavage events that are considered as central players in all apoptotic events in mammals. *Bcl-2* and *Bcl-x (L)* inhibit apoptosis, in part by blocking the release of cytochrome c from mitochondria. On the contrary, other family members, such as *bax* and *bad*, interfere with the activity of *bcl-2* by binding to them and generating a nonfunctional unit (14). 

Present study showed that TQ prevented apoptosis in seminiferous tubules treated with MTX through affecting mRNA expression levels of *p53, caspases 3, 8* and *9*, as well as the *Bax/Bcl-2* ratio. These data were confirmed by a clear decrease in the number of TUNEL positive apoptotic cells in MTX+TQ-treated mice. 

These results are consistent with previous report that showed combination of TQ and conventional chemotherapeutic drugs could produce greater therapeutic effect as well as reduce the toxicity of the latter (6). These data are also in parallel with the study of Nagi et al. (15), demonstrating that cyclophosphamide-induced cardiotoxicity in rats was attenuated through activity of TQ that results in reducing oxidative and nitrosative stress, as well as preserving the activity of antioxidant enzymes. In addition, TQ was shown to protect against MTX-induced testicular injury in male mice (9). 

## Conclusion

Combination of TQ with chemotherapeutic agents may provide a novel therapeutic approach. However, further research in animal models is warranted to obtain more conclusive evidence for the molecular basis of TQ action. Despite its therapeutic efficacy in tumor cell lines and animal models, the data on bioavailability and other pharmacokinetic parameters of TQ are still incomplete. 
